# The Potential for the Use of Edible Insects in the Production of Protein Supplements for Athletes

**DOI:** 10.3390/foods12193654

**Published:** 2023-10-03

**Authors:** Ewelina Zielińska, Urszula Pankiewicz

**Affiliations:** Department of Analysis and Food Quality Assessment, University of Life Sciences in Lublin, Skromna 8 Street, 20-704 Lublin, Poland; urszula.pankiewicz@up.lublin.pl

**Keywords:** banded cricket, edible insects, entomophagy, protein supplements, sports, athletes

## Abstract

Several types of proteins are used in athletes’ supplementation; nevertheless, given the problem of protein deficiency in the world and the growing need for ecological sources of protein, it is very interesting to study the quality of alternative protein sources, such as insect protein. This study investigated the nutritional value, micronutrient content, amino acid profile, and chemical score of banded cricket protein quality in the form of flour, defatted flour, and a protein preparation, as well as popular commercial protein supplements. In addition, in vitro digestion was performed, and the antiradical activity of the hydrolysates was compared. Generally, the defatted cricket flour was the most similar to commercial supplements regarding nutritional value because it contained 73.68% protein. Furthermore, the defatted flour was abundant in essential minerals, such as iron (4.59 mg/100 g d.w.), zinc (19.01 mg/100 g d.w.), and magnesium (89.74 mg/100 g d.w.). However, the protein preparation had an amino acid profile more similar to that of commercial supplements (total content of 694 mg/g protein). The highest antiradical activity against ABTS^·+^ was noted for the defatted flour (0.901 mM TE/100 g) and against DPPH^·^ for the cricket flour (2.179 mM TE/100 g). Therefore, cricket can be considered an organic protein source for the production of valuable protein supplements.

## 1. Introduction

Edible insects have undoubtedly become the trend of the last decade. This has manifested in the growing interest of researchers and the food industry in this topic. Specifically, after the report on edible insects as food and feed published in 2013 by the Food and Agriculture Organization of the United Nations [1], scientists began exploring the topic, which has resulted in many scientific publications year after year [2]. These, in turn, have contributed to the emergence of the new European Union Regulation 2015/2283 on novel foods [3]. In addition, the European Food Safety Authority (EFSA), based on numerous publications, published a scientific opinion on the safety of the first edible insects [4,5]. The number of insect farms and the range of insect products have also increased in recent years. The first products with insects on the European market were snacks, e.g., in the form of bars, but nowadays, products such as pasta, bread, drinks, and even ready meals (such as burgers or falafel) are offered [6,7,8,9].

Insect consumption is promoted for several reasons. The first is the high nutritional value of insects—they are a good source of complete protein. Moreover, they contain fat with a good fatty acid profile. Finally, in smaller amounts, we find carbohydrates or chitin, which act as dietary fiber in the human diet [6]. In addition to the nutritional value, the consumption of insects brings health-promoting value to our bodies. A wide range of biofunctional components (e.g., chitin, polyphenols, antioxidant enzymes, and antimicrobial peptides/proteins) is found in insects [10,11]. As a rich source of protein, insects are precursors of bioactive peptides with a wide range of activities—antimicrobial, antihypertensive, antioxidant, antidiabetic, anticancer, hepatoprotective, and hypocholesterolemic activities [6,11,12,13,14,15]. These properties suggest that the use of insects may prevent many civilization diseases. Moreover, a thorough literature review suggests that the bioactive potency of edible insect protein hydrolysates/peptides is similar to or higher than that of other dietary proteins (plants and animals) [11].

Another aspect is the environmental impact of insect farming. Generally, insects are considered an ecological protein. Insects produce less greenhouse gasses and have a higher feed conversion efficiency than conventional livestock. Moreover, insect feeds can be obtained from a wider range of plants than conventional livestock because agro-food industry waste, such as vegetable or fruit residues, can be used [1].

Overall, the advantages of using insects as food are obvious, but it is worth thinking about their specific use. Due to the excellent source of protein that they represent, they can be used in athletes’ nutrition. The nutrient quality of insect protein is prospective compared to that of casein and soy. The most edible insects appropriately provide the required essential amino acids [16]. Moreover, edible insects can be rich in vitamins and minerals, such as copper, iron, magnesium, manganese, phosphorous, selenium, and zinc, which are equally important in the diet of athletes [16,17]. Several new protein supplements have recently been introduced into the market, including energy bars enriched with insect flour. Further development of this market is probably imminent.

Other researchers have already raised the idea of using insects in the diet of athletes. An objective of a study conducted by Vangsoe et al. [18] aimed to compare the postprandial amino acid (AA) availability and AA profile in the blood after ingesting protein isolate from the lesser mealworm, whey isolate, and soy isolate. Significant increases in the blood concentration of essential amino acids (EAAs), branched-chain amino acids (BCAAs), and leucine were detected over a 120 min period for all protein supplements in this study. However, the blood concentrations of EAAs, BCAAs, and leucine peaked 60 min after both whey and soy protein consumption and 120 min after insect protein consumption. In addition, insect proteins were found to induce blood AA concentrations similarly to soy protein [18]. The differences in blood AA concentrations between whey, soy, and insect protein are due to the slower digestion of the insect protein. Another study investigated potential motivations for the acceptance of an energy protein bar with cricket flour among a group of selected Italian professional athletes and measured how an information intervention about the benefits of edible insects would affect acceptance. The protein content and curiosity about texture were the main drivers to taste the cricket energy bar, and male athletes were more likely than females to consume a bar. Moreover, an increase in willingness to taste was observed after information on the benefits of eating insects was provided [16]. In turn, Tang and Chung [19] studied sensory acceptance criteria for insects, such as the detection threshold (DT), consumer rejection threshold (CRT), and taste acceptance for mealworm powder added to protein shakes at various concentrations (DT, 2–8%; CRT and Liking, 3–15%). Moreover, consumers were divided into groups—the “no frame” group evaluated all samples in a blind condition. The “healthy frame” group and the “sustainable eating frame” group watched a video clip emphasizing the importance of healthy eating and sustainable eating, respectively, before sensory evaluation. The results proved to be very interesting because the mealworm CRT was identified only in the “no frame” group (14.6%). The other groups preferred samples with mealworms more than or equally to control samples at all concentration ranges. Additionally, the DT results suggested that information about a sustainable eating framework may cause consumers to be less sensitive to the flavor of mealworms and to view their higher concentration in protein shakes positively [19].

Ren, Yang, and Gu [20] investigated the factors influencing fitness enthusiasts’ willingness to purchase insect protein foods as fitness protein substitutes. According to this study, perceived value is one of the most important factors influencing their willingness to purchase insect food alternatives, such as protein products. The presence of insect protein products for athletes on the market would allow for the diversification of the available proteins on the market due to their origin and environmental impact [21]. 

The ongoing research on the use of insects in the diet of athletes, however, is sparse against the background of many works focusing on the use of insects in food technology. There is a lack of studies directly addressing popular supplements for athletes and comparing their basic characteristics. Furthermore, the studies conducted to date do not consider the various forms of insects but only the insect powder. However, the defatted powder or isolated protein can be used as nutritional supplements, thus requiring a more extensive assessment. 

The aim of our study was to verify the potential of edible insects to produce supplements for athletes by evaluating the quality of the banded cricket (*Gryllodes sigillatus*) flour, the defatted flour, and the protein preparation and compare them to commercially available protein supplements for athletes—the whey protein concentrate and the micellar casein. This particular insect species is a popular choice for breeding in Europe and is said to have the potential to be used as food and feed inside the European Union according to the EFSA [3].

## 2. Materials and Methods

### 2.1. Raw Materials

The crickets, *Gryllodes sigillatus* (Walker, Orthoptera: Gryllidae) (adult), were obtained from a commercial supplier from Poland. All individuals of these species were fasted for approximately 48 h to clear their gastrointestinal tract of residual food. Approximately 0.5 kg of the material was frozen and lyophilized for each species tested for further processing. 

### 2.2. Obtaining Insect Flour

The lyophilized insects were ground in a laboratory grinder (A11 basic, IKA, Warsaw, Poland) to obtain flour. Then, the flour was passed through a 20-mesh sieve to obtain a uniform particle size as previously reported [22].

### 2.3. Obtaining the Protein Preparation

The Girón-Calle, Alaiz, and Vioque method [23] was slightly modified to obtain the protein preparation [22]. For 1 h at room temperature, 0.2% NaOH (pH 11) was added to the insect flour at a ratio of 1:10 (*w*/*v*) and stirred. Next, the samples were centrifugated at 8000× *g* (MPW-351e, MPW, Warsaw, Poland) and the proteins were precipitated at the isoelectric point (pH 4.5). Next, centrifugation and the washing in distilled water of the obtained proteins were carried out. Afterwards, the protein preparations were lyophilized (Labconco, Kansas City, MO, USA) and kept at −18 °C. 

### 2.4. Defatting of Flour

The flour was defatted according to the Bußler et al. method [24] with modifications [22]. First, to remove the fat, the mixture of hexane and insect flour was stirred (magnetic stirrer MS 11 HS, WIGO, Jaszowice, Poland) for 2 h in a 5:1 hexane: flour ratio. The hexane was poured off, and the residual hexane was removed by evaporation overnight.

### 2.5. Calculation of Extraction Yield and Efficiency

The ratio of the weight of the protein preparation obtained to the weight of the flour used to extract the protein was used as the extraction yield [25]:

(1) $$Yield(\%)=\left(\frac{weight\;of\;protein\;preparation\;(g)}{weight\;of\;flour\;(g)}\right)\times 100,$$

The ratio of the protein content of the resulting protein preparation to the protein content of the insect flour determined by the Kjeldahl method was used as the extraction efficiency [25]:

(2) $$Extraction\;efficiency\;(\%)=\left(\frac{protein\;content\;in\;preparation\;(g)}{protein\;content\;in\;insect\;flour(g)}\right)\times 100,$$

### 2.6. Nutritive Value

The nutritional value (moisture, ash, fat, and protein content) was determined according to the Association of Official Agricultural Chemists (AOAC) methods [26]. The formula: 100—(weight in grams (protein + fat + ash + moisture) in 100 g) was used for the carbohydrates determination. The energy content was calculated using the conversion method [27].

### 2.7. Minerals Content

For mineral content determination, 0.5 g of the sample was weighed, 4 mL of nitric acid (V) was added, followed by mineralization in a microwave oven (Mars, Xpress, CEM). The mineralizates were quantitatively transferred to 10 mL volumetric flasks and made up to the mark with deionized water. The concentration of the mineral ions in the mineralizates was determined using flame atomic absorption spectrophotometry (FAAS, Solaar 939, Unicam) [28]. 

### 2.8. Determination of the Amino Acid Composition, Calculation of the Limiting Amino Acid Index and the Essential Amino Acid Integrated Index (EAAI)

The content of amino acids in the samples was determined with an automatic amino acid analyzer AAA-400 (INGO’s) by the Central Research Laboratory of the University of Life Sciences in Lublin. 

Protein quality was evaluated by the amino acid score, which is a calculation that compares the amount of the essential amino acid in a protein to the amount of that amino acid in a reference protein:

(3) $$Amino\;acid\;score=\frac{mg\;of\;amino\;acid\;in\;1\;g\;of\;the\;test\;protein}{mg\;of\;amino\;acid\;in\;1\;g\;of\;reference\;protein},$$

Moreover, the essential amino acid index (EAAI) was calculated based on the content of all essential amino acids compared to a reference protein, being values for human requirements in this case [29]. EAAI estimates the potential of using insects as a protein source for human consumption and a good source of amino acids in sports nutrition [30].

### 2.9. In Vitro Digestion

The simulated digestion process was based on the Monro et al. method [31]. The process was carried out without light at 37 °C with 100 rpm stirring (N-BIOTEK, NB-205, Pyeongcheon-ro, Bucheon-si, Gyeonggi-do, Republic of Korea). First, 1 g of the sample and 30 mL of water, 0.8 mL of 1 M HCl (to obtain a pH of 2.5), and 1 mL of pepsin (0.1 g/mL of 0.05 M HCl) were added to a test tube and incubated for 30 min for simulated gastric digestion. After this time, proceeding to simulated intestinal digestion, 2 mL of 1 M NaHCO_3_, 5 mL of 0.1 M phosphate buffer (pH = 6), and 4.35 mg of amyloglucosidase and 5 mL of 2.5% pancreatin (0.125 g/5 mL) were added. Next, the sample tube was filled to 55 mL with distilled water and incubated for 120 minutes. At the end of the digestion process, the enzyme activity was stopped by boiling the samples for 5 min, and the samples were centrifuged and used for further analyses.

### 2.10. ABTS^+^ (2,20-Azino-bis(3-ethylbenzothiazoline-6-sulfonic Acid) Radical) Scavenging Activity

Antiradical activity against ABTS^·+^ was determined as described by Re et al. [32] with slight modifications regarding the quantities of the antioxidant solution. First, the solution of ABTS^·+^ was diluted to reach the absorbance measures around 0.7 at 734 nm. A total of 2.95 mL of the ABTS^·+^ solution and 0.05 mL of the sample were mixed. The absorbance was measured (at 734 nm) after 3 min of the reaction with deionized water as a blank. The antiradical activity of the analyzed samples was calculated with the equation:

(4) $$Scavenging\;activity\;(\%)=\left[1-A_{sample}/A_{control}\right]\times 100,$$

where A sample is the absorbance of the sample and ABTS^·+^ mixture; A control is the absorbance value of the control (ABTS^+^ solution).

The results were expressed as Trolox equivalent antioxidant activity (TEAC) values (mM Trolox/100 g).

### 2.11. DPPH^·^ (2,2-Diphenyl-1-picrylhydrazyl Radical) Scavenging Activity

Antiradical activity against DPPH^·^ was determined as described by Brand-Williams, Cuvelier, and Berset [33], with slight modification. A 0.1 mL sample volume and 0.9 mL of 6 mM DPPH^·^ in 75% methanol were mixed. The absorbance was measured (515 nm) after 3 min of reaction with 75% methanol as a blank. The antiradical activity was calculated according to the equation:

(5) $$Scavenging\;activity\;(\%)=\left[1-A_{sample}/A_{control}\right]\times 100$$

where A sample is the absorbance value of the sample and DPPH^·^ mixture; A control is the absorbance value of the control (DPPH^·^ solution).

The results were expressed as Trolox equivalent antioxidant activity (TEAC) values (mM Trolox/100 g).

### 2.12. Statistical Analysis

All assays were performed in triplicate, and the data are presented as the means plus standard deviation. Statistical analyses were performed using a one-way analysis of variance, followed by the Tukey test using Statistica (version 13.0, StatSoft, Krakow, Poland). *p* < 0.05 was a statistically significant difference.

## 3. Results and Discussion

### 3.1. Nutritional Value and Mineral Content

Optimizing nutrient intake is key in supporting sports performance and aiding training adaptation. Protein requirements in athletes depend on the nature, duration and intensity of exercise, age, gender, and health status [34]. Nevertheless, the effect of exercise on nitrogen balance has been the subject of numerous studies that have confirmed increased protein consumption during exercise. Providing the body with sufficient protein protects muscles from injury, stimulates fat burning, and boosts muscle protein synthesis, especially when combined with strength training [34,35]. The primary distinction between sports nutrition products lies in the amount of protein they contain; however, the supplement’s total energy content should also be considered, which is affected by the amount of other nutrients, particularly fat. The defatted flour had as high a protein content (73.68 ± 0.62%) as commercial supplements (*p* < 0.05) (Table 1). Surprisingly, the protein preparation had a lower protein content (71.91 ± 0.5%) than the defatted flour. Bearing in mind the cricket protein extraction yield of 39.53 ± 1.5% using the proposed process and the extraction efficiency of 43.65 ± 1.7%, it is clear that protein extraction from crickets via the proposed method is not an economical option. The cricket flour was found to have 65.06 ± 0.73% protein, which is quite a high level. This flour had the highest fat content (17.53 ± 2.19%) of the tested forms and, therefore, the highest calorific value (448 ± 5.2 kcal). In turn, the protein preparation was characterized by a higher fat content (9.26 ± 0.19%) than the defatted flour (2.9 ± 0.33%). At the same time, the protein preparation contained the lowest amount of ash that can be considered mineral content. It is justified because minerals are lost during protein extraction. Flour and defatted flour (5.1 ± 0.13% and 5.72 ± 0.09%, respectively) contained more ash than the whey protein concentrate (4.2 ± 0.3%), but the micellar casein was found to be the most abundant in microelements (7.23 ± 0.03%). Based on these results, we can conclude that defatted flour is the best insect variant among the tested forms. Defatted flour is most similar to commercial protein supplements in terms of nutritional and calorific value. Simultaneously, it is much more abundant in ash and less in fat than the cricket protein preparation, which also has a high protein content of over 70%. However, the nutritional value of insects is quite difficult to analyze because it depends on many factors, e.g., the origin of the insect, stage of metamorphosis, diet, and environmental factors (temperature, humidity) [36]. An example is the different nutritional value of the banded cricket we used in our other studies from a different breeder—its protein content was higher (70%) than in the cricket used now [17]. It can be concluded that the different nutritional values of the same insect species may be due to different feed or breeding conditions used by the breeders.

Although insects are a good source of protein, nutrients, and minerals, it should be remembered that not all insects are safe to eat. Like other food ingredients from plants or animals, insects can cause allergies. Allergic reactions following insect consumption may be associated with the presence of allergenic proteins in them and cross-reactivity between ingested insects and taxonomically related species to which an individual has an existing allergy. This reaction may occur due to the phylogenetic relationship of insects to common allergen sources, such as crustaceans or house dust mites [37,38]. In the literature, we can find a few cases of reporting allergic reactions following the intentional consumption of insects. The species that caused allergic reactions were locusts, grasshoppers, silkworms, yellow mealworms (*Tenebrio molitor*), bee pupae and larvae, or moths [39].

Many micronutrients play a crucial role in energy metabolism. During strenuous physical activity, the rate of energy turnover in skeletal muscle can be increased by up to 20–100 times the resting rate. Prolonged, strenuous exercise performed regularly can also result in increased losses from the body or increased turnover rates, resulting in the need for increased dietary intake. Among the minerals, the much higher iron content in all of the insect forms tested is worth noting compared to commercial supplements (Table 2). There is no doubt that iron plays a crucial role in processes related to physical performance. Unfortunately, it is very often a deficient element. Iron is rightly associated with hemoglobin and oxygen transport in the blood. However, this is not the only role of iron. It is also involved in cellular respiration, helps to store oxygen in muscles in the form of myoglobin, is a cofactor for many enzymes, and is used by the immune system to fight pathogens. All of this makes iron important for maintaining health and high performance in athletes [40]. Another element in which the tested forms of cricket are more abundant than the commercial supplements is zinc. Zinc is involved in the metabolism of proteins, fats, and sugars, improves hormone production, enhances immunity, participates in DNA synthesis, and improves pancreatic function. The action related to hormone management and protein metabolism is a crucial aspect from an athlete’s perspective, as it helps to build quality muscle tissue and maintain normal cortisol levels, which can negatively affect muscles. In addition, zinc speeds up the body’s recovery after intense exercise, helps heal wounds, reduces soreness, and minimizes the risk of specific debilitating injuries [41,42]. Moreover, the flours contain significantly more potassium than commercial supplements. For example, the defatted flour contains twice as much potassium as whey protein concentrate. Potassium belongs to the group of electrolytes, i.e., minerals involved in controlling the body’s water balance. Potassium is also involved in numerous enzymes and significantly controls protein and glycogen synthesis. Potassium deficiency can cause muscle weakness, cramps, irregularity, muscle breakdown, or impaired glucose tolerance, so an adequate supply of this element in the diet of athletes is significant [43]. The magnesium content of the tested flours is quite similar to that of the commercial supplements, and at the same time, they contain less sodium than whey protein concentrate. Magnesium stabilizes the nervous system, is essential for the cardiovascular system’s normal function, helps maintain healthy bones, and increases performance [43]. In turn, the studied insect forms are much less abundant in calcium than commercial supplements of milk origin. Calcium binding to troponin C is fundamental to muscle contraction and theoretically affects athletes’ performance. In addition, calcium loss increases during exercise, mainly through sweat [43]. Athletes often use micronutrient supplements to correct mineral deficiencies, improve immune function, enhance recovery, or optimize performance [43]. The presence of minerals in athletes’ protein supplements is an added advantage.

### 3.2. Amino Acid Content and the Chemical Score of Protein Quality

To assess protein quality, knowing its amino acid composition is crucial. It is important to know that banded cricket is a complete protein source (Table 3). Amino acids are a significant part of every athlete’s diet. Supplementation is often necessary because the body cannot produce essential amino acids independently, and its food supply is sometimes insufficient. The evaluation of amino acid (AA) profiles in the different insects’ forms and the protein supplements revealed that the highest content of total essential AA was found in the whey protein concentrate—339.9 ± 0.93 mg/g protein. In turn, the protein preparation was characterized by the highest total essential AA among studied insects’ forms—272.5 ± 0.78 mg/g protein. The essential AA in the whey protein concentrate constituted 45% of all amino acids and the micellar casein—40%. In turn, in the insect flours, it was 36% and the protein preparation value was similar to in the micellar casein—39%. Flour and defatted flour had a lower total essential AA content than the reference protein proposed by WHO/FAO/UNU. Nevertheless, the amino acid content can vary, as can the overall nutritional value of the insect, which was discussed earlier. The total essential AA content in the banded cricket protein studied in our earlier manuscript was estimated at 304.6 ± 5.76 mg/g protein [17]. It is a big difference; therefore, the breeding conditions should be optimized to obtain the highest possible protein content with the desired amino acid profile. Such an effort would make it possible to obtain a protein that precisely responds to the requirements of athletes. Such variability in the nutritional value of insects or the amino acid composition of their protein is problematic because we cannot unequivocally assess the quality of a selected insect species—it will vary according to its origin. However, this can also be an advantage because we can easily modify the nutritional value of insects. 

People who train need BCAA in particular. They are the building blocks for muscle proteins, including leucine, isoleucine, and valine. They form about 1/3 of muscle protein. Their appropriate supplementation increases training capacity and speeds up regeneration processes so an athlete can train longer and more intensively. In addition, they inhibit muscle catabolism, positively affect growth hormone synthesis, and have an anabolic effect. Increasing exercise intensity and duration increases the amino acid oxidation rate, especially once glycogen reserves have been depleted. A lack of adequate protein may reduce the athlete’s exercise capacity [44]. The highest concentration of BCAA was determined in the whey protein concentrate (175.3 ± 0.47 mg/g protein), but among the studied insect forms, the protein preparation was characterized as the highest value (132 ± 0.29 mg/g protein). The BCAA in the whey protein concentrate constituted 23% of all amino acids, in the micellar casein—20.5%, and in all of the studied insect forms—19%. Thus, we can conclude that the BCAA content of the used banded cricket is more similar to the micellar casein than the whey protein concentrate.

Protein quality can also be evaluated by the chemical score (CS), which compares the essential amino acid content in the studied sample to the content in the reference protein. The essential amino acid index (EAAI) is based on the content of all essential amino acids compared to a reference protein, being valued for human requirements in this case [29]. The amino acid with the lowest CS is the limiting amino acid in the studied protein. For example, phenylalanine is the limiting amino acid for whey protein concentrate and cricket flours; for the micellar casein—leucine; and the protein preparation—valine (Table 4). The calculated essential amino acid index (EAAI) of all insect forms was lower than that of whey protein concentrate (140.68%) and micellar casein (130.61%). The most comparable value was found for the protein preparation (116.61). However, digestibility data must be considered for more detailed insight into insect protein quality since digestibility is not included in determining the EAAI. Many researchers have studied the digestibility of insect protein and calculated it from in vitro studies, ranging from 76% to 98%, indicating that insects might have a high nutritional value [45].

### 3.3. Antioxidant Activity of Protein Hydrolysates

A stimulus such as physical exertion (especially over a long period) is undoubtedly a stress factor for the body. Whether we are involved in the sport as a recreational or professional activity, our organism has a greater need for antioxidant compounds during increased exertion. This is because free oxygen radicals are formed in the body during exercise [46]. Oxidative stress reflects an imbalance between the production of reactive oxygen species and adequate antioxidant defense. This adverse state can damage cells and tissue components and is involved in various physiopathological conditions, including ageing, exercise, inflammatory, cardiovascular, and neurodegenerative diseases, and cancer [47]. 

In addition to nutritional effects, protein’s effect on the body’s antioxidant potential is also significant, determining the ability to inactivate free radicals, which is essential given the increased oxidative stress in sports. Increased protein catabolism processes in sport (so-called exercise-induced catabolism) result from mechanical and chemical damage to protein structures caused, for example, by free radicals. All high-protein products are good sources of peptides, but the activity of the obtained peptides is not the same. Many studies have shown that insects are a source of bioactive peptides with antioxidant properties and many other properties, e.g., antimicrobial, antihypertensive, and antidiabetic [11,13,14]. Moreover, the antioxidant activity of peptides from insect protein was higher than that from other food products known as antioxidants, e.g., orange juice [14,15]. It is since the antioxidant peptides obtained during the digestion of insect proteins have stronger antioxidant properties than the compounds in the juice, mainly vitamin C.

**Figure 1:**
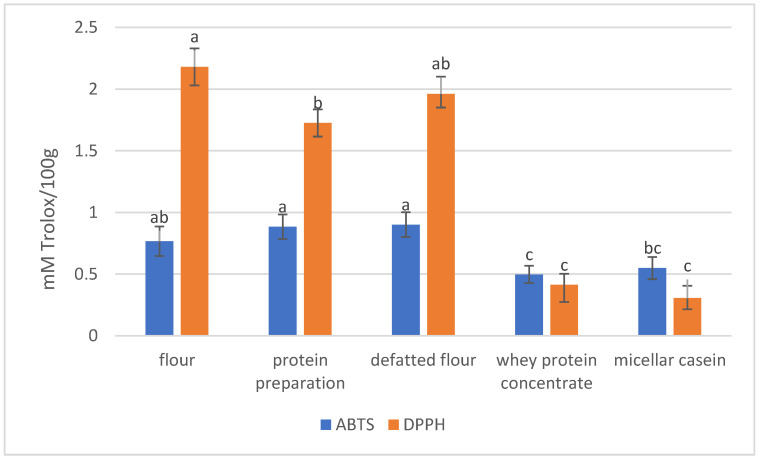
Antioxidant activity of hydrolysates against ABTS^·+^ and DPPH^·^. Different letters in the same radical type (ABTS or DPPH) indicate a significant difference (*p* < 0.05).

We performed a simulated digestion of the test samples to determine the antioxidant potential of the hydrolysates. The antioxidant activity was expressed as free radical-scavenging activity, and the difference between the studied insect forms and commercial supplements is significant (Figure 1). A higher difference is observed in the case of DPPH^·^ scavenging activity, where the insect flour hydrolysates’ activity was even five times higher than that of commercial supplements hydrolysates (*p* < 0.05). The highest antiradical activity against DPPH^·^ was noted for the hydrolysates obtained from the cricket flour (2.18 mM TE/100 g) and defatted flour (1.96 mM TE/100 g). The whey protein concentrate and the micellar casein hydrolysates were only 0.41 and 0.3 mM TE/100 g, respectively. In the case of the antiradical activity against ABTS^·+^, the difference was insignificant; however, the hydrolysates obtained from the tested forms of insects (flour 0.776 mM TE/100 g, defatted flour 0.8901 mM TE/100 g, and protein preparation 0.884 mM TE/100 g) were characterized by a higher activity than the commercial supplements hydrolysates (whey protein concentrate 0.497 mM TE/100 g and micellar casein 0.549 mM TE/100 g), as well as strong antioxidant orange juice (0.4 mM TE/100 g). These values are similar to the popular strong antioxidant ascorbic acid (1 mM TE/100 g) [48].

This noticeable difference in the antioxidant properties of commercial supplements hydrolysates and insects is due to the difference in the origin of the protein and, thus, its structure. Antioxidant peptides are oligopeptides, including 2–20 amino acids in their structure, and the types of amino acids in the peptide sequence significantly impact their activity [49,50]. Although the relationship between the antioxidant and structural properties of peptides has not been explained in detail, generally, peptides with low molecular weight and hydrophobic and aromatic amino acids in the structure have a better antioxidant activity [49,51]. Therefore, we can surmise that insect proteins are precursors to more of these peptides than the milk proteins that whey protein and casein supplements are derived from.

Protein supplements for athletes are currently differentiated only by the quantity and quality of protein; the aspect of antioxidant properties is not considered [52]. The results indicate the high antioxidant activity of insect hydrolysates, which may be an added value when used to produce supplements for athletes. Due to these properties, insects can be classified as high-protein and health-promoting products [11].

## 4. Conclusions

The data suggest that insect flour or the protein extracted from insects may be a potential future alternative to most proteins in athletes’ nutrition. However, to select one of the insect variants studied as the most suitable for the production of supplements, it is necessary to consider all the issues raised by the analyses. First, the protein preparation was found to have the most similar amino acid profile to the commercial supplements. Nevertheless, the defatted insect flour had a higher protein content than the protein preparation. The protein extraction yield (39.53 ± 1.5%) and extraction efficiency (43.65 ± 1.7%) from crickets using the proposed method are insufficient to make the process economical. Additionally, the protein extraction process would be more complicated, longer, and costly, and this aspect is also important because it determines the ability to compete with other supplements. Moreover, the defatted flour is more abundant in minerals, which is its added value. With the above, the defatted flour seems to be the most favorable form of insects among the three tested from the nutritional and economic perspectives.

However, the limitations of this study should be noted. Due to the high variability in the nutritional value of insects dictated by many factors, the comparison is precisely for this batch of banded crickets and these supplements. Therefore, the possible use of insects for the production of supplements must be followed by the optimization of insects’ breeding conditions to obtain the same quality of insects, which would facilitate the production process. However, there is a need for further research to assess the usefulness of insect protein in athletes’ nutrition because it appears promising.

## Figures and Tables

**Table 1:** Nutritional value of tested forms of insects and commercial supplements.

	Protein (%)	Fat (%)	Ash (%)	Carbohydrates (%)	Moisture (%)	Energy 100 g (kcal)
flour	65.06 ± 0.73 ^c^	17.53 ± 2.19 ^a^	5.1 ± 0.13 ^c^	7.57 ± 0.68 ^b^	4.75 ± 0.22 ^d^	448 ± 5.2 ^a^
defatted flour	73.68 ± 0.62 ^a^	2.9 ± 0.33 ^c,d^	5.72 ± 0.09 ^b^	10.03 ± 0.54 ^a^	7.66 ± 0.31 ^c^	361 ± 6.1 ^d^
protein preparation	71.91 ± 0.5 ^b^	9.26 ± 0.19 ^b^	3.16 ± 0.18 ^e^	8.36 ± 0.44 ^b^	7.31 ± 0.08 ^c^	404 ± 4.9 ^b^
whey protein concentrate	75 ± 0.5 ^a^	4.2 ± 0.3 ^c^	4.49 ± 0.07 ^d^	11 ± 0.48 ^a^	12.41 ± 1.36 ^a^	382 ± 4.4 ^c^
micellar casein	75 ± 0.44 ^a^	0.9 ± 0.08 ^d^	7.23 ± 0.03 ^a^	8 ± 0.39 ^b^	9.61 ± 0.34 ^b^	340 ± 5.7 ^e^

^a,b,c,d,e^ Different letters in the same column indicate a significant difference (*p* < 0.05).

**Table 2:** Mineral composition of tested forms of insects and commercial supplements (mg/100 g d.w.).

	Minerals Content (mg/100 g d.w.)					
	Fe	Ca	Zn	Mg	K	Na
flour	3.86 ± 0.1 ^c^	223.78 ± 0.34 ^c^	19.29 ± 0.23 ^a^	79.69 ± 0.17 ^d^	1248 ± 9.7 ^b^	325.7 ± 0.56 ^d^
defatted flour	4.59 ± 0.15 ^b^	207.74 ± 0.25 ^d^	19.01 ± 0.18 ^a^	89.74 ± 0.15 ^b^	1529 ± 6.7 ^a^	346.86 ± 0.54 ^c^
protein preparation	7.23 ± 0.21 ^a^	16.08 ± 0.11 ^e^	11.98 ± 0.12 ^b^	18.58 ± 0.11 ^e^	343.4 ± 2.0 ^e^	564.49 ± 0.36 ^a^
whey protein concentrate	2.75 ± 0.09 ^d^	674.9 ± 0.54 ^b^	0.9 ± 0.03 ^d^	86.64 ± 0.26 ^c^	684.5 ± 2.1 ^c^	526.52 ± 0.17 ^b^
micellar casein	1.0 ± 0.02 ^e^	1921 ± 8.2 ^a^	8.07 ± 0.1 ^c^	105.4 ± 0.54 ^a^	584.2 ± 1.12 ^d^	149.5 ± 0.14 ^e^
Recommended daily intake (mg/day) (FAO, 2004)	7.5–58.8	100–1300	3–14	220–260	4700	1500

^a,b,c,d,e^ Different letters in the same column indicate a significant difference (*p* < 0.05).

**Table 3:** The amino acid content of tested forms of insects and commercial supplements (mg/g protein).

Amino Acids	Tested Forms of Cricket (*Gryllodes sigillatus*)			Tested Commercial Forms of Supplements		WHO/FAO/UNU Reference Protein ^1^	
	Flour	Defatted Flour	Protein Preparation	Whey Protein Concentrate	Micellar Casein	(mg/g Protein)	(mg/kg Body Mass/Day)
Isoleucine *^B^	28.6 ± 0.07 ^e^	34.4 ± 0.06 ^c^	31.2 ± 0.05 ^d^	49.0 ± 0.2 ^a^	38.3 ± 0.09 ^b^	30	20
Leucine *^B^	43.1 ± 0.1 ^e^	49.7 ± 0.08 ^d^	62.0 ± 0.1 ^c^	81.1 ± 0.12 ^a^	72.7 ± 0.12 ^b^	59	39
Lysine *	35.3 ± 0.09 ^e^	39.4 ± 0.05 ^d^	52.4 ± 0.11 ^c^	73.5 ± 0.15 ^a^	61.7 ± 0.08 ^b^	45	30
Methionine *	15.4 ± 0.04 ^e^	15.9 ± 0.04 ^d^	21.6 ± 0.08 ^a^	16.8 ± 0.07 ^c^	21.3 ± 0.07 ^b^	16	10
Cysteine *	10.5 ± 0.05 ^d^	11.9 ± 0.04 ^c^	13.9 ± 0.06 ^b^	16.7 ± 0.07 ^a^	4.1 ± 0.02 ^e^	6	4
Total sulphur a.a. **	25.9 ± 0.09 ^d^	27.8 ± 0.08 ^c^	35.5 ± 0.14 ^a^	33.5 ± 0.14 ^b^	25.4 ± 0.09 ^e^	22	14
Phenylalanine *	20.2 ± 0.11 ^d^	23.0 ± 0.07 ^c^	32.4 ± 0.09 ^b^	23.0 ± 0.05 ^c^	37.6 ± 0.07 ^a^	30	25
Tyrosine	30.2 ± 0.08 ^d^	36.8 ± 0.1 ^b^	33.7 ± 0.11 ^c^	19.9 ± 0.05 ^e^	40.4 ± 0.09 ^a^		
Total aromatic a.a. ***	50.4 ± 0.19 ^d^	59.8 ± 0.17 ^c^	66.1 ± 0.2 ^b^	42.9 ± 0.1 ^e^	78 ± 0.16 ^a^	30	25
Threonine *	23.8 ± 0.12 ^e^	27.4 ± 0.12 ^d^	34.1 ± 0.15 ^b^	51.3 ± 0.12 ^a^	32.7 ± 0.1 ^c^	23	15
Valine *^B^	33.5 ± 0.1 ^e^	38.4 ± 0.1 ^d^	38.8 ± 0.14 ^c^	45.2 ± 0.15 ^b^	49.0 ± 0.12 ^a^	39	26
Total essential a.a.	199.9 ± 0.68 ^e^	228.2 ± 0.56 ^d^	272.5 ± 0.78 ^c^	339.9 ± 0.93 ^a^	313.3 ± 0.67 ^b^	242	165
Total BCAA	105.2 ± 0.27 ^e^	122.5 ± 0.24 ^d^	132 ± 0.29 ^c^	175.3 ± 0.47 ^a^	160 ± 0.33 ^b^		
Histidine	14.0 ± 0.07 ^d^	17.7 ± 0.15 ^b^	16.8 ± 0.13 ^c^	13.0 ± 0.07 ^e^	21.3 ± 0.08 ^a^		
No essential a.a.							
Aspartic acid	49.7 ± 0.15 ^e^	58.5 ± 0.21 ^c^	74.2 ± 0.21 ^b^	84.2 ± 0.21 ^a^	53.0 ± 0.09 ^d^		
Serine	25.1 ± 0.09 ^e^	30.0 ± 0.15 ^d^	30.9 ± 0.08 ^c^	35.2 ± 0.07 ^b^	41.9 ± 0.12 ^a^		
Glutamic acid	71.4 ± 0.17 ^e^	80.0 ± 0.17 ^d^	100.9 ± 0.24 ^c^	138.6 ± 0.27 ^b^	163.6 ± 0.21 ^a^		
Proline	33.9 ± 0.12 ^d^	37.3 ± 0.09 ^c^	29.0 ± 0.1 ^e^	42.1 ± 0.09 ^b^	79.7 ± 0.2 ^a^		
Glycine	31.5 ± 0.09 ^b^	35.6 ± 0.06 ^a^	31.4 ± 0.07 ^b^	10.7 ± 0.07 ^d^	13.8 ± 0.05 ^c^		
Alanine	45.4 ± 0.16 ^b^	54.2 ± 0.12 ^a^	41.2 ± 0.12 ^c^	38 i.3 ± 0.08 ^d^	23.7 ± 0.04 ^e^		
Arginine	38.4 ± 0.11 ^c^	44.9 ± 0.09 ^b^	49.5 ± 0.08 ^a^	16.1 ± 0.03 ^e^	26.1 ± 0.07 ^d^		
Total a.a.	550 ± 1.72 ^e^	635.1 ± 1.7 ^d^	694 ± 1.92 ^c^	754.7 ± 1.87 ^b^	780.9 ± 1.62 ^a^		

* essential amino acids, ** Methionine + cysteine, *** Phenylalanine + tyrosine, a.a. = amino acids, ^1^ (WHO, 2007), ^B^ branched-chain amino acids BCAA. ^a,b,c,d,e^ Different letters in the same row indicate a significant difference (*p* < 0.05).

**Table 4:** Chemical score of protein quality of tested forms of insects and commercial supplements.

Amino Acids	Chemical Score of Protein Quality (CS) (%)				
	Flour	Defatted Flour	Protein Preparation	Whey Protein Concentrate	Micellar Casein
Isoleucine	95.33	114.67	104.00	163.33	127.67
Methionine	96.25	99.38	135.00	105.00	133.13
Leucine	73.05	84.24	105.08	137.46	123.22
Threonine	103.48	119.13	148.26	223.04	142.17
Lysine	78.44	87.56	116.44	163.33	137.11
Phenylalanine	67.33	76.67	108.00	76.67	125.33
Valine	85.90	98.46	99.49	115.90	125.64
EAAI	85.68	97.16	116.61	140.68	130.61
Amino acid limiting	Phenylalanine	Phenylalanine	Valine	Phenylalanine	Leucine

## Data Availability

The data presented in this study are available on request from the corresponding author.
